# Theory and Design of the Community for successful ageing (ComSA) program in Singapore: connecting BioPsychoSocial health and quality of life experiences of older adults

**DOI:** 10.1186/s12877-019-1277-x

**Published:** 2019-10-09

**Authors:** Su Aw, Gerald C. H. Koh, Chuen Seng Tan, Mee Lian Wong, Hubertus J. M. Vrijhoef, Susana Concordo Harding, Mary Ann B. Geronimo, Zoe J. L. Hildon

**Affiliations:** 1 Saw Swee Hock School of Public Health, National University of Singapore, Tahir Foundation Building, 12 Science Drive 2, #08-01, Singapore, 117549 Singapore; 2 Department of Patient and Care, Maastricht University Medical Centre, Maastricht, The Netherlands; 3 Department of Family Medicine, Vrije Universiteit Brussel, Brussel, Belgium; 4 Tsao Foundation, Singapore, Singapore; 5 Bloomberg School of Public Health, The John Hopkins University, Maryland, USA; 6 Faculty of Public Health and Policy, Department of Global Health and Development, London School of Hygiene and Tropical Medicine, Keppel street, London, WC1E 7HT UK

**Keywords:** BioPsychoSocial programs, Quality of life, Holistic conceptualization of health, Older adults

## Abstract

**Background:**

Despite the emphasis on holistic health promotion in community programs for older people, few studies explicitly consider how BioPsychoSocial (BPS) health elements are interconnected and function to improve Quality of Life (QoL). The Community for Successful Ageing (ComSA) program in Singapore focuses on Community Development (CD) initiatives for older people, accounting for BPS theory in its design and content. Biological (B) health is conceived as physiological and cognitive functioning and related biological self-care; Psychological (P) health as feelings of life satisfaction, and Social health (S) as perceived social support and civic engagement.

Furthermore, three overlapping sub-constructs are theorized to connect these elements. Namely Bio-Psychological (BP) health in terms of self-perceptions of ageing; the Psycho-Social (PS) aspects of interpersonal communication; and the Socio-Communal (SC) health in terms of civic engagement. BPS health is conceived as distinct from QoL, defined as composed of control, autonomy, self-realisation and pleasure (measured by CASP-19) of the older person.

We examined 1) interconnections of BPS constructs and related sub-constructs and 2) their associations with QoL to inform a practical, applied program theory.

**Methods:**

A baseline survey (*n* = 321) of program participants (Mean = 70 years, SD = 8.73). All continuous variables were binarized as ‘high’ if the scores were above the median. Multivariate logistic regression was used to assess 1) the adjusted effect of each BPS construct on CASP-19, and 2) the odds of scoring high on one BPS construct with the odds of scoring high on a related sub-construct (e.g. B and BP health).

**Results:**

The strongest relationship with QoL was markedly with BP self-perceptions of ageing (OR = 4.07, 95%CI = 2.21–7.49), followed by P life satisfaction (OR = 3.66, 95%CI = 2.04–6.57), PS interpersonal communication (OR = 2.42, 95%CI = 1.23–4.77), SC civic engagement (OR = 1.94, 95%CI = 1.05–3.57), and S social support (OR = 1.89, 95%CI = 1.06–3.38). Core B, P and S health were closely associated with their sub-constructs.

**Conclusion:**

ComSA CD is tightly coupled to its proposed program theory. It offers classes to improve B self-care and BP self-perceptions of ageing, group-based guided autobiography to improve P life-satisfaction and PS interpersonal communication, and community initiatives that encourage seniors to solve community issues. This holistic approach is likely to enhance ageing experiences and QoL.

## Background

Current empirical and theoretical models emphasize that successful ageing is not just about maintaining biological function for as long as possible. The emphasis has shifted instead to *adapting* well [1–3] to age-related bio-physical and cognitive declines, potentially shrinking social network and quality of interactions, as well as psychological threats to the self from role transitions. This shift is consistent with existing thinking that emphasizes the resilient and dynamic nature of ageing and human development [4–6], as well as proactive and asset-based [7] health promotion.

To increase adaptive capability [5, 8], many community programs target different aspects of BioPsychoSocial (BPS) health [9–13], to promote Quality of Life (QoL) of community-dwelling older adults early, before or on the onset of their retirement [7]. However, few among them explicitly theorize and test how the different aspects of BPS health can interrelate and promote QoL. We therefore examine the ComSA Community Development or ‘ComSA CD’ program [14], as a case study to address this gap. The program uses a community development approach [15], by first improving participants’ BPS health, then galvanizing them to act on community issues that influence their health, all of which in turn are hypothesized to promote QoL.

### Integrated BPS theory on successful ageing

Traditionally, QoL of older people has been measured using health-related proxies, which conflates influences on QoL with QoL itself. Using a needs satisfaction model posited by Hyde et al. (2003) [16], the current program theory and analyses argues that QoL at older ages is theoretically distinct from health. Rather it is underpinned by the more active and reflexive aspects of ageing experiences [17] and dependant on whether core needs of the older person are satisfied from these experiences. These include - control (ability to actively intervene in one’s environment), autonomy (to be free from unwanted interference of others), self-realization (of activities meaningful to the person) and pleasure (in lived activities), and can be measured using the CASP-19 scale [16]. These are particularly relevant in the Third Age - the period between retirement and Fourth Age, where individuals are physically more independent to pursue leisure and personal interests.

While Biological functional limitations are consistently associated with a reduction in CASP-19 QoL [18–20], good Psychological [21–24] and Social health [25–27] can offset the effect of declining biological health [5, 28] and are closely related with QoL [28–34]. Lived experiences of ageing show that older people can continue to maintain high-quality fulfilling lives by using inner psychological and external social resources to adapt and overcome their illness [4–6]. Consequently, QoL is hypothesized to be catalysed by psychosocial aspects of health.

**Fig. 1:**
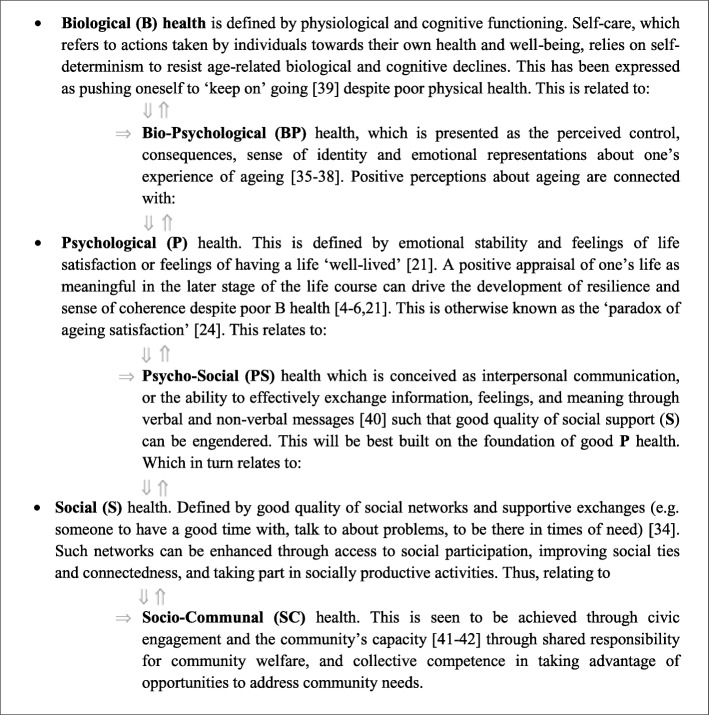
Definitions of BioPsychoSocial health and their interconnections. Proposed Pathway of how the six BioPsychoSocial Health constructs important for Quality of Life at old age are *associated* with one another. Pathway is derived from literature and existing empirical studies. Bio-Psychological (BP), Psycho-Social (PS) and Social-Communal (SC) sub-constructs are indented to show how they *extend* and *connect* the existing BPS constructs, demarcated by the bi-directional arrow

To promote BPS health and QoL of community-dwelling older adults living in the Singapore estate of Whampoa, ComSA CD targeted three core BPS health constructs and three additional but commonly accepted health-related constructs that were theorized to connect them. These include the Bio-Psychological (BP) *self-perceptions of ageing* [35–39], the Psycho-Social (PS) *interpersonal communication* skills [40] and the Socio-Communal (SC) *civic engagement* [41, 42]. All six BPS health constructs are defined, and their interconnections theorized in Fig. 1.

The program was commissioned in 2014, as part of a City of All Ages Initiative [43] by the Ministerial Committee on Ageing to create age-friendly neighborhoods in Singapore. The neighborhood of Whampoa was selected as a pilot-program site, as 23.5% of its residents were 65 years and above, compared to the average of 11% in other estates [44].

### Connecting BPS theory and program design

Based on an iterative process of matching known theoretical constructs onto programming approaches [5, 45], ComSA CD was designed to provide three program components, tightly coupled to the program theory.

‘Self-Care of Older Adults’ (**SCOPE**) targets B and BP health through 16 weeks of lessons about ageing well (ageing perceptions) and self-care. Self-care refers to the state where older people are able to promote or maintain their health and functioning, and self-care programs delivered in the community have been found to increase physical exercise, decrease in health distress, and improve self-rated health [46–48]. Four areas of self-care targeted in SCOPE, including healthy eating, exercise, health monitoring and chronic disease management, and communication with health professionals.

‘Guided-Autobiography’ **(GAB)** targets P and PS health, through 8 weeks of structured reminiscence about their life experiences in a group therapy format, which has been shown to improve life satisfaction, ego-integrity, sense of mastery, positive well-being and social integration [23, 49].

‘Sharing Wellness and Initiatives Interest Group’ (**SWING**) targets S and SC health, using an eight week participatory workshop to foster critical community assessment and thinking on community solutions, which are known operational domains for community development and capacity-building [15, 41]. After 8 weeks, all SWING groups are combined into a larger group which meet monthly in dialogue sessions, as well as social activities. The program framework therefore is built around the larger community development concept while embedding a BPS health promotion approach.

**Fig. 2:**
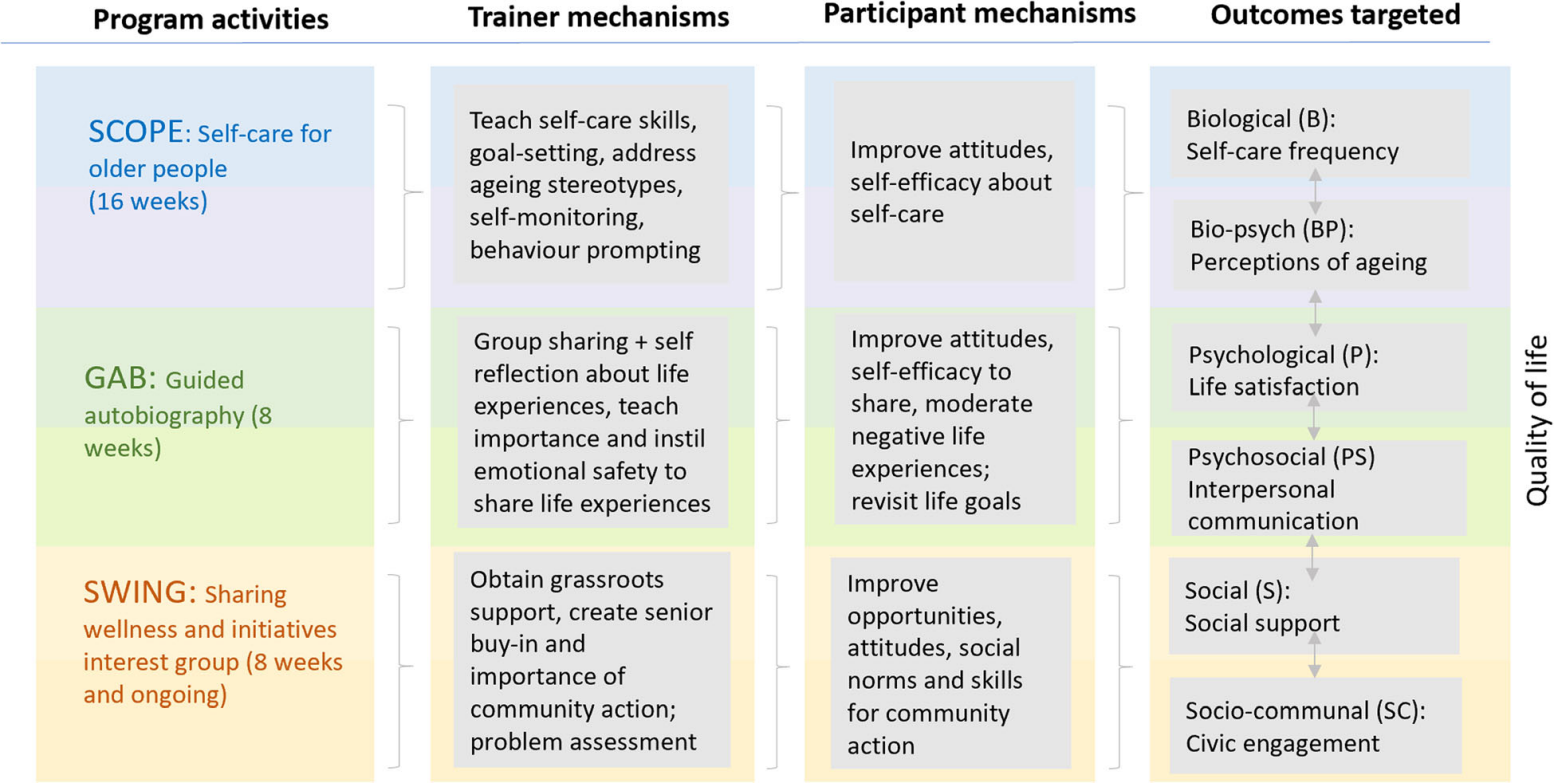
Connecting BPS Theory and Program Design in ComSA CD. Design of the ComSA CD program. Program *components* (SCOPE, GAB, SWING) are mapped onto respective BioPsychoSocial target *outcomes*, and hypothesized to work using respective *mechanisms* as the change pathway, as demarcated by the arrows. Trainer mechanisms refer to implementation strategies used by program trainers based on literature review of similar programs. Participant mechanisms refer to individual-level determinants of change, which were the most significant change barriers from ComSA CD formative assessment

Figure 2 lists the trainer and participant mechanisms that are adopted in SCOPE, GAB and SWING. These were hypothesized to improve BPS outcomes, based on a community assessment [8] conducted with older adults and community leaders in Whampoa, assessing barriers to change for the program. These barriers were mapped to relevant change techniques used by trainers in similar BPS programs [9–13] and informed by social cognitive theory [50].

### Aim and objectives

We therefore aim to test the program’s BPS theory at baseline to specifically validate the content of the ComSA CD program and better inform its implementation. Towards this aim, our specific objectives are to verify that:

1. The core BPS health constructs were associated with their sub-constructs (according to Fig. 2).
2. BPS constructs and their sub-constructs were associated with QoL.

## Method

### Study backdrop

This study is part of a larger longitudinal mixed-methods evaluation (2016–2018). Herein we elaborate and test the program’s BPS theory by validating the interconnections between core BPS constructs and sub-constructs as well as their associations with QoL. In a linked qualitative study, we explore the program’s implementation from an organizational perspective [51], while a mixed-methods outcome evaluation is in preparation.

### Study design

We adopted a quantitative cross-sectional analysis from a baseline survey (*n* = 321) of participants to verify the relationships proposed in Fig. 2.

### Sampling and recruitment method

Residents living in the Whampoa neighbourhood and older than 50 years old, were invited by Tsao Foundation and the research team to join the program through community sign-up booths, presentations with community partners, flyers and banners. The program was promoted across 13 Whampoa community sites, including nine resident committee centres, one religious’ institution, two senior activity centres and one community club.

Individuals who agreed to join the program were invited by a contracted survey company to complete a baseline survey within 3 weeks of starting the program - at home or in the community at a location they desired. Those who rejected the program (not-exposed) were also invited to complete the survey. The total response rate for the survey was 73.8%. Informed consent was obtained before survey administration.

### Data collection

The CASP-19 questionnaire [16] was used to measure four domains of QoL (control, autonomy, self-realization, and pleasure), which are specific to older people, and distinct from health. The measure has been validated across cultural contexts [52–55]. All items in the questionnaire were translated from English to Mandarin, Malay, Tamil. We pilot-tested with 5–10 members of the respective ethnic groups in Whampoa by asking them to interpret each questionnaire item using their own words and comment on its ease of understanding. We refined the items based on their feedback before administration.

For B self-care, 18 behavioural items were self-constructed to measure: following a healthy diet (9 items), communication with the doctor (4 items), exercise (3 items) and disease management (3 items). A final subdomain score of ‘1’ was given if all subdomain items scored ‘yes’ and ‘0’ if otherwise. The range of the self-care score is from 0 to 4.

BP self-perceptions was measured using a 16-item questionnaire [35] that assessed the perceived control (e.g. ‘whether I continue living life to the full depends on me’), consequence (e.g. ‘as I get older, I get wiser’), emotional representations (e.g. ‘I worry about the effects that getting older may have on my relationships with others’) and identity of ageing (e.g. ‘I feel my age in everything I do’). Negative items were scored from 0 to 5 on a Likert scale while positive items were reverse-coded; total scores ranged from 0 to 90.

P life satisfaction was measured using the 8-item Life Satisfaction Index [56] which assessed ‘acceptance and contentment’ with life in old age. Each item was scored from 0 to 3 on a Likert Scale; total scores range from 0 to 24. For PS Interpersonal communication, 4 behavioural items were self-constructed to measure the frequency which the older person engaged their social networks to cope their problems, rather than used avoidance-based coping. These included ‘use my past experiences to discuss the present issues’, ‘decided to avoid the problem and think about it later’, ‘talked through the issues with people I trust’ and ‘do what I can to understand and accept the problem.’ Each item was scored from 1 to 4 on a Likert scale; total scores ranged from 1 to 16.

S social support was measured using the 3-item social subscale from the BPS Risk Questionnaire [5] that assessed the number of friends living in the participant’s neighbourhood whom they could ‘see or hear from at least once a month’, ‘feel at ease with that you can talk about private matters’ and ‘feel close to such that you could call on them for help’. Each item was scored 0 to 5 on a Likert scale; total scores ranged from 0 to 15.

SC civic engagement was assessed through 7 self-constructed behavioural items to measure whether in the past 6 months, one volunteered in the community, interacted with one’s neighbors, helped one’s neighbors, attended community meetings, raised community issues, solved community problems, and organized community activities. Response to these items were yes (scored as ‘1’) or no (scored as ‘0’). Total scores ranged from 0 to 7.

In general, higher scores indicated better levels of the outcome, except for self-perceptions of ageing, which was reverse-coded, as higher scores indicated more negative perceptions. There was good reliability for all scales (α > 0.70) except for the life satisfaction scale which had moderate reliability (α = 0.60). Sociodemographic variables, presence of longstanding illness and functional problems (e.g. problems with seeing, hearing and communicating, activities of daily living and getting around) were also collected.

### Methods of analysis

All continuous variables were binarized as ‘high’ if the scores were above the median and ‘low’ if otherwise, except for the ageing perceptions scale which was binarized in the opposite direction. Ethnicity was binarized into ‘Chinese’ and ‘Other’ (Indian, Malay and others). To describe participants’ baseline characteristics (Table 1), we reported the percentages for all categorical variables, and median with interquartile range for the continuous age variable.

**Table 1:** Survey Participant Characteristics at Baseline (*N* = 321) *

Socio-demographics		
Mean Age (SD)		70 (8.73)
Female (%)		77.9
Ethnicity (%)		
Chinese		81.0
Indian/Malay		19.0
Primary Education and Above (%)		73.5
Own a House (%)		90.7
Living Alone (%)		19.6
At least one longstanding Illness (%)		70.1
Functional Problems (%)		63.9
BPS health (%)		
B	Frequency of Self-Care	
	High	60.8
BP	Perceptions of Ageing	
	High	44.9
P	Life Satisfaction	
	High	42.1
PS	Interpersonal Communication	
	High	28.7
S	Social Health	
	High	43.9
SC	Civic Engagement	
	High	36.1
Primary Variable (%)		
CASP19 Quality of Life		
High		50.2

*continuous = Kruskal Wallis test, categorical = chi-square association, high = above the median score

To verify that the 6 BPS health domains at baseline are associated with baseline QoL, the bivariate relationship of each BPS variable with QoL was examined using logistic regression (model 1 of Table 2). Next, to see the independent effect of each BPS variable, we included all 6 BPS variables in a multivariate model (model 2 of Table 2). This multivariate model adjusted for age, gender, ethnicity, education, housing, living alone, functional problems and longstanding illness, as they are known confounders of QoL in the literature. There was no missing data for the variables included in the analysis. Age was binarized into 75 and below, or above 75, for the purpose of the logistic regression.

**Table 2:** Logistic Regression predicting Higher Quality of Life at Baseline (N = 321)

	Model 1 (unadjusted bivariate)		Model 2 (adjusted multivariate) *	
			R^2^ = 0.31	
	OR (95%CI)	*p*	OR (95%CI)	*p*
B: Frequency of Self-Care				
Low	ref		ref	
High	1.90 (1.21–2.99)	0.01	1.47 (0.80–2.67)	0.21
BP: Perceptions of Ageing				
Low	ref			
High	7.49 (4.54–12.34)	< 0.01	4.07 (2.21–7.49)	< 0.01
P: Life Satisfaction				
Low	ref			
High	5.63 (3.46–9.19)	< 0.01	3.66 (2.04–6.57)	< 0.01
PS: Interpersonal Communication				
Low	ref			
High	4.55 (2.65–7.82)	< 0.01	2.42 (1.23–4.77)	0.01
S: Social Support				
Low	ref			
High	2.77 (1.76–4.37)	< 0.01	1.89 (1.06–3.38)	0.03
SC: Civic Engagement				
Low	ref			
High	2.58 (1.61–4.14)	< 0.01	1.94 (1.05–3.57)	0.03

*Adjusted for age, gender, ethnicity, education, housing, living alone, functional problems and longstanding illness

As the first item of the CASP19 scale e.g. (“My age prevents me from doing the things I would like to”) overlaps with the perceptions of ageing items, a sensitivity analysis between the BP perception of ageing scale and the CASP19 scale where the overlapping item was dropped, was performed.

To test the interrelationships of BPS health in Fig. 2, logistic regression was used to assess the odds of scoring high on one construct with the odds of scoring high on a related construct (Table 3). We reported the odds ratio (OR) and its corresponding 95% confidence interval (95%CI). *P*-values less than 0.05 are considered significant. The analysis was conducted using the STATA13 software.

**Table 3:** Odd Ratios between Biopsychosocial Constructs at Baseline (*N* = 321)

	BP: High Ageing perceptions	P: High Life Satisfaction	PS: High Interpersonal communication	S: High Social Support	SC: Civic Engagement
	OR (95%CI)	OR (95%CI)	OR (95%CI)	OR (95%CI)	OR (95%CI)
B: High Self-care	1.72 (1.04–2.83) *				
BP: High Ageing perceptions*		3.80 (2.35–6.15)			
P: High Life Satisfaction			2.29 (1.40–3.74)		
PS: High Interpersonal communication				2.31 (1.42–3.80)	
S: High Social Support					2.05 (1.29–3.25)

* Between B self-care and BP positive ageing consequence subscale

## Results

### Participants’ baseline characteristics

Referring to Table 1, the majority of participants were female, of Chinese ethnicity, had at least primary education, owned a house, lived alone, reported at least one longstanding illness and reported having problems with at least one daily activity of living. The mean age was 70 years old (SD = 8.73).

### Association with QoL

Referring to model 1 of Table 2, all 6 BPS indicators had a significant bivariate association with CASP-19 QoL at baseline. However, in the multivariate-adjusted model (model 2), high QoL was associated with all BPS components except B self-care.

The strongest relationship with QoL was markedly with BP self-perceptions of ageing (OR = 4.07, 95%CI = 2.21–7.49), followed by P life satisfaction (OR = 3.66, 95%CI = 2.04–6.57), PS interpersonal communication (OR = 2.42, 95%CI = 1.23–4.77), SC civic engagement (OR = 1.94, 95%CI = 1.05–3.57), and S social support (OR = 1.89, 95%CI = 1.06–3.38). Sensitivity analysis revealed that the association between BP self-perceptions of ageing and CASP-19 remained strong (OR = 3.51, 95% CI = 1.91–6.45), even after removing the first item in CASP-19.

### Interrelationship of BPS health constructs and sub-constructs

Referring to Table 3, all health constructs and sub-constructs were positively associated with one another in the proposed directions in Fig. 2. Association between BP self-perceptions of ageing and high P life satisfaction was the strongest (OR = 3.80, 95%CI = 2.35–6.15). This was followed by associations between having high PS interpersonal communication and high S social support (OR = 2.31, 95%CI = 1.42–3.80). Having high P life satisfaction was also associated with having good PS interpersonal communication (OR = 2.29, 95%CI = 1.40–3.74), as was having a high quality of S social support and SC civic engagement (OR = 2.05, 95%CI = 1.29–3.25).

High B self-care frequency was only associated with the subscale of positive ageing consequence, e.g. ‘as I get older I feel that I get wiser’ (OR = 1.72, 95%CI = 1.04–2.83), and not with 4 other subscales of positive control, negative control and consequence, ageing identity and emotional representations of ageing.

## Discussion

The aim of this study was to verify the program theory that 1) the core BPS health constructs were associated with their sub-constructs, and that 2) all six BPS health constructs were all associated with QoL. To increase adaptive capability [5, 8], many community programs target different aspects of BioPsychoSocial (BPS) health [9–13] among community-dwelling older adults, early or on the onset of retirement, However, few among them explicitly theorize and test how the different aspects of BPS health can interrelate and promote QoL.

The significant bivariate relationship between QoL with all 6 BPS health constructs supports our theory that holistic BPS health promotion is related with QoL of older adults. However, in the face of limited resources, BPS programs could prioritize targeting BP self-perceptions of ageing and P life satisfaction, as these constructs were most strongly associated with QoL in the multivariate analysis. These associations remained present even after adjusting for longstanding illness, functional problems, and sociodemographic factors at baseline. Thus, they endorse the buffering or ‘lifting’ effect of good P and S health against declining B health, which is reflected in the ‘resiliency’ phenomenon, whereby older people are able to experience good QoL despite living with health-related adversity [4, 28, 29].

The strongest associations among the BPS constructs were between BP self-perceptions of ageing and P life satisfaction. This finding could be explained by Erikson [21] and Butler [22] who posit that older people gain a sense of continuity, meaning and wisdom in later life, through constructing and maintaining a positive and satisfying life narrative. The use of these positive life narratives may improve perceptions about ageing, in terms of growing wiser - expressed in the item ‘as I grow old, I get wiser’ in Sexton’s subscale of positive ageing consequence [35].

One interesting finding was that B self-care frequency was not associated with the negative perceptions of ageing (in terms of identity, emotional representation, consequences/ control), and only with perceptions about the positive consequence of ageing. This finding might suggest that to motivate B self-care, there is a need to go beyond targeting negative ageing perceptions (e.g. about declining health), to promoting optimism and things to look forward to ageing experiences. For example, Wurm and Benyamini (2013) found in a longitudinal survey of older adults in Germany that optimism modified the effect of negative self-perceptions of ageing on health. Older adults who expected negative consequences such as physical declines in ageing but were nevertheless optimistic and hopeful about the future, had better physical functioning at 3 year follow-up [57].

Lastly, the association between P life satisfaction with PS interpersonal communication points to the importance of psychological health in influencing communication of the older person with others, and in turn the quality of social support. Relatedly, an ethnographic study conducted within Whampoa showed that older people who were confident in managing social interactions were more likely to expand their social networks as compared to those who were fearful of social interactions and preferred to “comfort zone alone” [8]. These individuals may benefit from building up their confidence through participating in the P guided autobiography component of the program, before exsperiencing the S SWING component.

### Strengths and limitations

One limitation of our study was the self-selecting sample, as we recruited participants who were invited by Tsao Foundation to join the program. We attempted to address this by adjusting for the various sociodemographic variables in the regression analyses. The cross-sectional study design also limits knowledge about the directionality of associations. Nevertheless, there is currently no study that has simultaneously examined the empirical associations of these 6 BPS constructs with CASP-19 QoL, and their interconnections. By comparing the relative strength of their associations, this study contributes to programming knowledge on which BPS health assets to prioritize in relation to QoL.

## Conclusion

ComSA CD is tightly coupled to its proposed program theory. It offers classes to improve B self-care and BP self-perceptions of ageing, group-based guided autobiography seeking to improve P life-satisfaction and PS interpersonal communication, and activities that connect and encourages seniors to solve community issues. This holistic approach is likely to promote positive experiences of ageing and QoL. Regardless of BPS theory, successful implementation of the program is necessary to elicit necessary change mechanisms (see Fig. 2) among participants. We therefore explore the program’s implementation from the organizational perspective in a linked qualitative study [51], to provide lessons learnt on program delivery.

## Data Availability

The datasets used and/or analysed during the current study are available from the corresponding author on reasonable request.

## Abbreviations

B: Biological
BP: Bio-Psychological
BPS: BioPsychoSocial
CASP: Control, Autonomy, Self-realization, Pleasure
CI: Confidence Interval
ComSA: Community for Successful Ageing
GAB: Guided Autobiography
OR: Odds Ratio
P: Psychological
PS: Psycho-Social
QoL: Quality of life
S: Social
SC: Socio-Communal
SCOPE: Self Care of the Older Person
SWING: Sharing Wellness and Initiatives Interest Group

## References

[1] Marty M, Clara B (2015). Successful aging and its discontents: a systematic review of the social gerontology literature. Gerontologist.

[2] Bowling A, Dieppe P (2005). What is successful ageing and who should define it?. BMJ Br Med J.

[3] Fry PS (1992). Major social theories of aging and their implications for counselling concepts and practice: a critical review. Couns Psyschologist.

[4] Harris PB (2008). Another wrinkle in the debate about successful aging: the undervalued concept of resilience and the lived experience of dementia. Int J Aging Hum Dev.

[5] Hildon Z, Tan CS, Shiraz F, Ng WC, Deng X, Choon GH (2018). The theoretical and empirical basis of a BioPsychoSocial ( BPS ) risk screener for detection of older people ’ s health related needs , planning of community programs , and targeted care interventions. BMC Geriatr.

[6] Van Kessel G (2013). The ability of older people to overcome adversity: a review of the resilience concept. Geriatr Nurs (Minneap).

[7] Kahana E, Kahana B, Eun J (2014). Proactive approaches to successful aging : one clear path through the Forest. Gerontology.

[8] Aw Su, Koh Gerald, Oh Yeon Ju, Wong Mee Lian, Vrijhoef Hubertus J.M., Harding Susana Concordo, Geronimo Mary Ann B., Lai Cecilia Yoon Fong, Hildon Zoe J.L. (2017). Explaining the continuum of social participation among older adults in Singapore: from 'closed doors' to active ageing in multi-ethnic community settings. Journal of Aging Studies.

[9] Collins CC, Benedict J (2006). Evaluation of a community-based health promotion program for the elderly: lessons from seniors CAN. Am J Health Promot.

[10] Clark F, Jackson J, Carlson M, Chou C-P, Cherry BJ, Jordan-Marsh M (2012). Effectiveness of a lifestyle intervention in promoting the well-being of independently living older people: results of the well elderly 2 randomised controlled trial. J Epidemiol Community Health.

[11] Buijs R, Ross-Kerr J, Cousins SO, Wilson D (2003). Promoting participation: evaluation of a health promotion program for low income seniors. J Community Health Nurs.

[12] Lee E-KO, Yoon H, Lee J, Yoon J, Chang E (2012). Body-mind-Spirit practice for healthy aging. Educ Gerontol.

[13] Figueira H a., Figueira a. a., Cader S a., Guimaraes a. C, De Oliveira RJ, Figueira J a (2012). Effects of a physical activity governmental health programme on the quality of life of elderly people. Scand J Public Health.

[14] Tsao foundation. Community Development 2014. http://tsaofoundation.org/what-we-do/comsa/community-development (accessed August 4, 2016).

[15] Laverack G, Labonte R (2000). A planning framework for community empowerment goals within health promotion. Health Policy Plan.

[16] Hyde M, Wiggins RD, Higgs P, Blane DB (2003). A measure of quality of life in early old age: the theory, development and properties of a needs satisfaction model (CASP-19). Aging Ment Health.

[17] Higgs P, Hyde M, Wiggins R, Blane D (2003). Researching quality of life in early old age: the importance of the sociological dimension. Soc Policy Adm.

[18] Wiggins R, Higgs P, Hyde M, Blane D (2004). Quality of life in the third age: key predictors of the CASP-19 measure. Ageing Soc.

[19] Howel D (2012). Interpreting and evaluating the CASP-19 quality of life measure in older people. Age Ageing.

[20] Hyde M, Higgs P, Wiggins RD, Blane D (2015). A decade of research using the CASP scale: key findings and future directions. Aging Ment Health.

[21] Erikson EH. Childhood and society. 2nd ed: WW Norton & Company; 1963.

[22] Butler RN (1963). The life review: an Intepretation of reminiscence in the aged. Psychiatry.

[23] Bohlmeijer E, Roemer M, Cuijpers P, Smit F (2007). The effects of reminiscence on psychological well-being in older adults: a meta-analysis. Aging Ment Health.

[24] Kunzmann U, Little TD, Smith J (2000). Is age-related stability of subjective well-being a paradox? Cross-sectional and longitudinal evidence from the Berlin aging study. Psychol Aging.

[25] Young Y, Frick KD, Phelan EA (2009). Can successful aging and chronic illness coexist in the same individual? A multidimensional concept of successful aging. JMDA.

[26] McMunn A, Nazroo J, Wahrendorf M, Breeze E, Zaninotto P (2009). Participation in socially-productive activities, reciprocity and wellbeing in later life: baseline results in England. Ageing Soc.

[27] Siegrist J, Wahrendorf M (2009). Participation in socially productive activities and quality of life in early old age: findings from SHARE. J Eur Soc Policy.

[28] Hildon Z, Smith G, Netuveli G, Blane D (2008). Understanding adversity and resilience at older ages. Sociol Health Illn.

[29] Hildon Z, Montgomery SM, Blane D, Wiggins RD, Netuveli G (2010). Examining resilience of quality of life in the face of health-related and psychosocial adversity at older ages: what is “right” about the way we age?. Gerontologist..

[30] Adamczuk J, Szymona-Pałkowska K, Robak JM, Rykowska-Górnik K, Steuden S, Kraczkowski JJ (2015). Coping with stress and quality of life in women with stress urinary incontinence. Menopausal Rev.

[31] Zaninotto P, Falaschetti E, Sacker A (2009). Age trajectories of quality of life among older adults: results from the English longitudinal study of ageing. Qual Life Res.

[32] Netuveli G, Wiggins RD, Hildon Z, Montgomery SM, Blane D (2006). Quality of life at older ages: evidence from the English longitudinal study of aging (wave 1 ). J Epidemiol Community Heal.

[33] Liao J, Brunner EJ (2016). Structural and functional measures of social relationships and quality of life among older adults: does chronic disease status matter?. Qual Life Res.

[34] Litwin H, Kimberly JS (2014). Confidant network types and well-being among older Europeans. Gerontologist.

[35] Sexton E, King-Kallimanis BL, Morgan K, Mcgee H (2014). Development of the brief ageing perceptions questionnaire (B-APQ): a confirmatory factor analysis approach to item reduction. BMC Geriatr.

[36] Levy BR, Slade MD, Kunkel SR, Kasl SV (2002). Longevity increased by positive self-perceptions of aging. J Pers Soc Psychol.

[37] Wurm Susanne, Tomasik Martin J., Tesch-Römer Clemens (2008). Serious health events and their impact on changes in subjective health and life satisfaction: the role of age and a positive view on ageing. European Journal of Ageing.

[38] Levy BR, Ph D, Myers LM (2004). Preventive health behaviors influenced by self-perceptions of aging. Prev Med (Baltim).

[39] Shiraz* F, Su* A, Vrijhoef HJ, Hildon ZJ-L. Exploring the loads-levers-lifts model of successful ageing: examining the processes of bio-psycho-social adaptation in a multi-ethnic Asian older population. Prep Gerontol. n.d.

[40] Brooks WD, Heath RW. Speech communication: Brown & Benchmark; 1993.

[41] J a M, Bowen GL. Community resilience : a social organization theory of action and change, vol. 2009. p. 245–65.

[42] Bowen GL, Martin JA, Mancini J, Nelson JP (2000). Community capacity: antecedents and consequences. J Community Pract.

[43] Ministry of Family and Social Development. City for All Ages project to develop urban solutions for ageing 2014. https://app.msf.gov.sg/Press-Room/City-for-All-Ages-project (accessed August 4, 2016).

[44] Housing & Development Board (2013). Public housing in Singapore, residents’ profile, housing satisfaction and preferences.

[45] Huber M, Knottnerus JA, Green L, van der Horst H, Jadad AR, Kromhout D (2011). How should we define health?. BMJ.

[46] Jonker AAGC, Comijs HC, Knipscheer KCPM, Deeg DJH (2009). Promotion of self-management in vulnerable older people: a narrative literature review of outcomes of the chronic disease self-management program (CDSMP). Eur J Ageing.

[47] Foster G, Taylor SJC, Eldridge S, Ramsay J, Griffiths CJ. Self-management education programmes by lay leaders for people with chronic conditions. Cochrane database Syst Rev. 2007. 10.1002/14651858.CD005108.pub2.www.cochranelibrary.com. 10.1002/14651858.CD005108.pub2 17943839

[48] Nolte S, Osborne RH (2013). A systematic review of outcomes of chronic disease self-management interventions. Qual Life Res.

[49] Pinquart M, Forstmeier S (2012). Effects of reminiscence interventions on psychosocial outcomes: a meta-analysis. Aging Ment Health.

[50] Bandura A (2001). Social cognitive theory: an agentic perspective. Annu Rev Psychol.

[51] Aw S, Koh CH, Tan CS, Wong ML, Vrijhoef HJ, Harding SC, et al. Exploring the implementation of the Community for Successful Ageing (ComSA) program in Singapore: lesson learnt on program delivery for improving BioPsychoSocial health. BMC Geriatr. 2019. 10.1186/s12877-019-1271-3. 10.1186/s12877-019-1271-3 PMC6820954 31664899

[52] Tai-Yin W, Wei-Chu C, Kuan-Liang K, Wai-Kuen W, Jen-Pei L, Shih-Ting C (2013). Quality of life (QOL) among community dwelling older people in Taiwan measured by the CASP-19, an index to capture QOL in old age. Arch Gerontol Geriatr.

[53] Kim Gyu Ri, Netuveli Gopalakrishnan, Blane David, Peasey Anne, Malyutina Sofia, Simonova Galina, Kubinova Ruzena, Pajak Andrzej, Croezen Simone, Bobak Martin, Pikhart Hynek (2014). Psychometric properties and confirmatory factor analysis of the CASP-19, a measure of quality of life in early old age: the HAPIEE study. Aging & Mental Health.

[54] Sim J, Bartlam B, Bernard M (2011). The CASP-19 as a measure of quality of life in old age: evaluation of its use in a retirement community. Qual Life Res.

[55] Sexton E, King-Kallimanis BL, Conroy RM, Hickey A (2013). Psychometric evaluation of the CASP-19 quality of life scale in an older Irish cohort. Qual Life Res.

[56] James O., Davies Ann D. M., Ananthakopan S. (1986). The Life Satisfaction Index - Well-being: Its Internal Reliability and Factorial Composition. British Journal of Psychiatry.

[57] Wurm S, Benyamini Y (2014). Optimism buffers the detrimental effect of negative self-perceptions of ageing on physical and mental health. Psychol Health.

